# Prevention of alcohol, drugs and tobacco during the COVID-19-pandemic – consequences and inequalities in Swedish municipalities

**DOI:** 10.1093/eurpub/ckac129.007

**Published:** 2022-10-25

**Authors:** U Owen, E Lindström

**Affiliations:** 1 Living Conditions and Lifestyles, Public Health Agency of Sweden, Östersund, Sweden; 2 Living Conditions and Lifestyles, Public Health Agency of Sweden, Solna, Sweden

## Abstract

**Background and objectives:**

The COVID-19 pandemic and measures to prevent the spread of the virus challenged public health practice at the local and regional level in Sweden. The objective of this study was to follow-up how local preventive ADT prevention (alcohol, drugs, and tobacco) in Sweden was affected during 2020-2021.

**Methods:**

All Swedish municipalities (N = 290) were included in surveys on how the pandemic affected local ADT prevention. Response rates ranged between 82 and 91 percent. Quantitative data were analysed with reference to socioeconomic and demographic conditions. Qualitative data were analysed thematically.

**Results:**

A majority of the municipalities reported a decrease in ADT prevention, especially aimed at groups such as parents, children, and young people. There was no correlation between the decrease in municipal ADT prevention and socio-demographic conditions. A majority of the municipalities reported that activities were adapted, often with a digital approach. Adaptation of ADT prevention was less common in smaller municipalities and municipalities where residents had lower levels of education and lower incomes. An increase in activities, as a consequence of measures to prevent the spread of the virus, was more common in larger municipalities and municipalities with a greater proportion of residents with higher educational backgrounds and higher incomes.

**Conclusions:**

ADT prevention carried out by municipalities in Sweden was initially deeply affected by the COVID-19 pandemic and by measures to prevent the spread of the virus. However, activities were adapted over time, mainly with a digital approach. The ability to adapt differed depending on the sociodemographic conditions of the municipalities. Follow-up studies on ADT prevention and the consequences of the digital approach during 2021 will be presented at the conference.

